# Bone marrow stem cells-derived extracellular matrix is a promising material

**DOI:** 10.18632/oncotarget.21683

**Published:** 2017-10-09

**Authors:** Xiaoyan Wang, Guanghua Chen, Chao Huang, Hualei Tu, Jilong Zou, Jinglong Yan

**Affiliations:** ^1^ Department of Orthopedics, The Second Affiliated Hospital of Harbin Medical University, Harbin 150086, P.R. China; ^2^ Key Laboratory of the Ministry of Education, Ministry of Myocardial Ischemia, Harbin 150086, P.R. China; ^3^ Burn Department, The Fifth Hospital of Harbin, Harbin 150086, P.R. China; ^4^ Department of Orthopedics, The First Affiliated Hospital of Harbin Medical University, Harbin 150001, P.R. China

**Keywords:** extracellular matrix, BMSCs, tissue engineering, scaffold, bone regeneration

## Abstract

The extracellular matrix(ECM), which is primarily composed of collagens and proteoglycans, plays a key role in cell proliferation, differentiation, and migration and interactions between cells. In this study, we produced chitosan/gelatin/bone marrow stem cells-derived extracellular matrix(C/G/BMSCs-dECM) scaffolds via lyophilization and cross-linking, and chitosan/gelatin(C/G) scaffolds were used as controls. For the C/G/BMSCs-dECM scaffolds, the average pore size was 289.17 ± 80.28 μm; the average porosity was 89.25 ± 3.75%; the average compressive modulus was 0.82 ± 0.07 MPa; and the average water uptake ratio was 13.81 ± 1.00. *In vitro*, the C/G/BMSCs-dECM scaffolds promoted bone marrow stem cells(BMSCs) attachment and proliferation. Moreover, improved osteogenic differentiation was observed for these scaffolds. Thus, C/G/BMSCs-dECM is a promising material for bone tissue engineering.

## INTRODUCTION

Every year, millions of people develop bone defects for a variety of reasons, and the use of scaffolds for tissue engineering is a promising approach for the treatment of these defects [1]. Scaffolds composed of hydroxyapatite and tricalcium phosphate have been applied, and various products have been used commercially. However, the ability of these scaffolds to promote osteogenesis is not satisfactory; thus, researchers added osteogenic factors, such as vascular endothelial growth factor(VEGF) and transforming growth factor beta-1(TGF-β1), to cells via transfection, which were then incorporated into the scaffolds [2]. A functional scaffold should be osteoconductive, degradable and bioactive [3], but improving bioactivity through cell transfection is inefficient. Therefore, organic materials are needed for tissue engineering.

The extracellular matrix (ECM) is a fibrillar basement network of secreted proteins that plays a key role in cell proliferation, differentiation, and migration and the interactions between cells [4]. In *vivo*, the ECM is initially produced by cells and subsequently formed into a three-dimensional network [5], making it a suitable material for scaffolds. Decellularized organs derived from animals and humans have been made into scaffolds [6–8]; however, potential pathogen transmission limits their application. The fabrication of ECM on porous scaffolds has become increasingly popular, and several studies have shown good outcomes [9–12]. Briefly, cells are cultured with scaffolds for several days such that they adhere to the walls of the scaffolds and secrete ECM. Then, the scaffolds are decellularized using physical or chemical methods, and scaffolds coated with ECM are obtained. However, collagens and proteins are damaged during decellularization. Liming Wang extracted intact bone marrow stem cells-derived ECM (BMSCs-dECM) and used it to form scaffolds for cartilage regeneration [13]. This method retains the bioactivity of the ECM. Based on our previous experience, a portion of the ECM is lost when an ECM suspension is lyophilized. Moreover, the mechanical strength of ECM scaffolds is low [13]; therefore, scaffolds made entirely of BMSCs-dECM cannot be used for bone tissue engineering.

Chitosan is the only osteogenic cationic polysaccharide of natural origin [14], while gelatin is anionic. Satisfactory mechanical properties can be obtained with scaffolds composed of chitosan and gelatin, and these scaffolds have been used for bone tissue engineering [14–17]. Theoretically, the incorporation of ECM into chitosan and gelatin scaffolds may improve the mechanical properties of ECM scaffolds and the bioactivity and osteogenic capability of C/G scaffolds. In addition, ECM loss through lyophilization will not occur.

Stem cell differentiation is the key process of osteogenesis, adipogenesis and chondrogenesis. Moreover, previous studies have confirmed that the nanotopographical geometry, feature size and height can influence the phenotype and, eventually, the function of cells [18–20]. Hence, the scaffold structure is also a key factor in bone tissue engineering.

In this study, we constructed C/G/BMSCs-dECM scaffolds and investigated the feasibility of their application in bone tissue engineering.

## RESULTS

**Figure 1:**
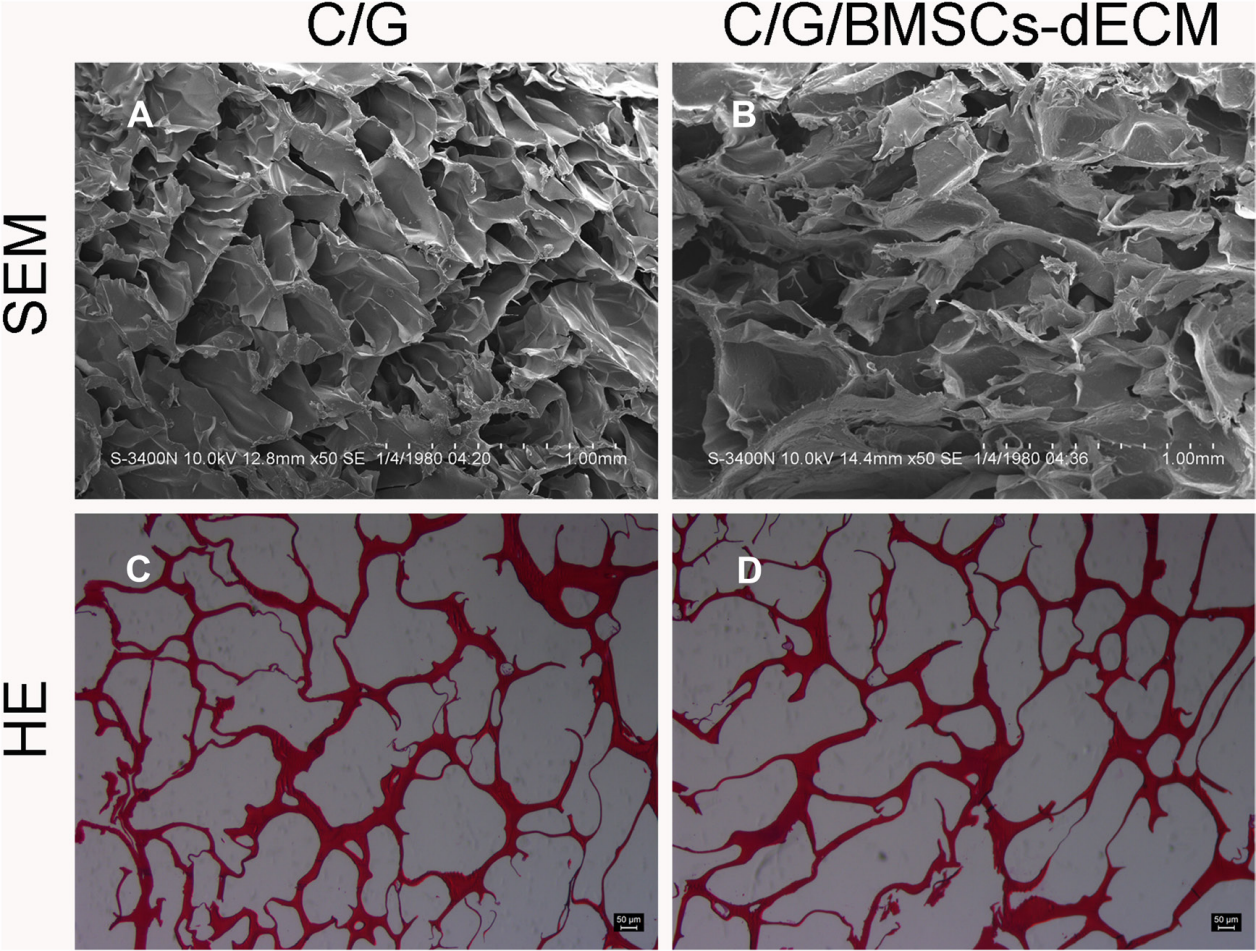
Internal structure Both types of scaffolds were test by SEM (**A**, **B**) and HE (**C**, **D**), which shared similar internal structure, connective pore and thin wall.

We obtained white and porous scaffolds via lyophilization, and two scaffold types shared similar surface and internal structures; specifically, the scaffolds had open pores, thin walls and a connective interior (Figure 1).

For the C/G/BMSCs-dECM scaffolds, the average pore size was 289.17 ± 80.28 μm, the average porosity was 89.25 ± 3.75%, the average water uptake ratio was 13.81 ± 1.00, and the average compressive modulus was 0.82 ± 0.07 MPa (Table 1). For the C/G scaffolds, the average pore size was 227.17 ± 44.37 μm, the average porosity was 88.48 ± 2.28%, the average water uptake ratio was 11.07 ± 0.87, and the average compressive modulus was 1.10 ± 0.11 MPa (Table 1).

**Table 1:** Caracteristics of C/G/BMSCs-dECM and C/G scaffolds

characteristics	C/G/BMSCs-dECM scaffolds	C/G scaffolds
Pore diameter (μm)	289.17 ± 80.28	227.17 ± 44.37
Porosity (%)	89.25 ± 3.75	88.48 ± 2.28
Water uptake ratio	13.81 ± 1.00	11.07 ± 0.87
Compressive modulus (MPa)	0.82 ± 0.07	1.10 ± 0.11

**Figure 2:**
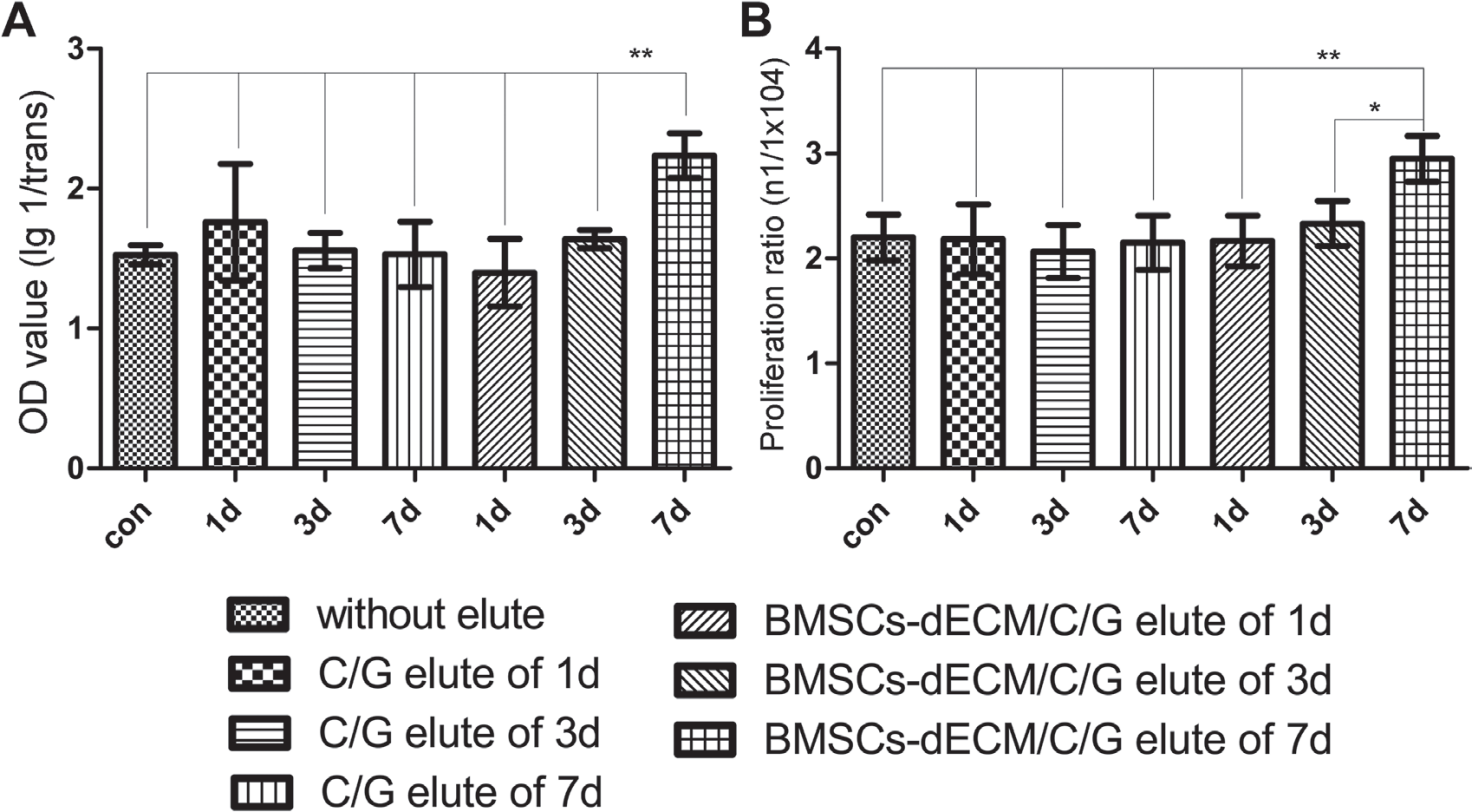
Cell activity and proliferation after 3d Values are mean ± standard error of the mean (*n* = 6). ^**^P < 0.05, significant change with respect to control group.

The cell counting kit-8 (CCK-8) assay results showed that the OD value associated with elution from the C/G/BMSCs-dECM scaffolds at 7 days was higher than that of the other groups, and there were no significant differences among the other 6 groups (Figure 2A). In addition, the proliferation ratio of the cells associated with the C/G/BMSCs-dECM scaffold elution at 7 days was also higher than that of the other groups (Figure 2B).

**Figure 3:**
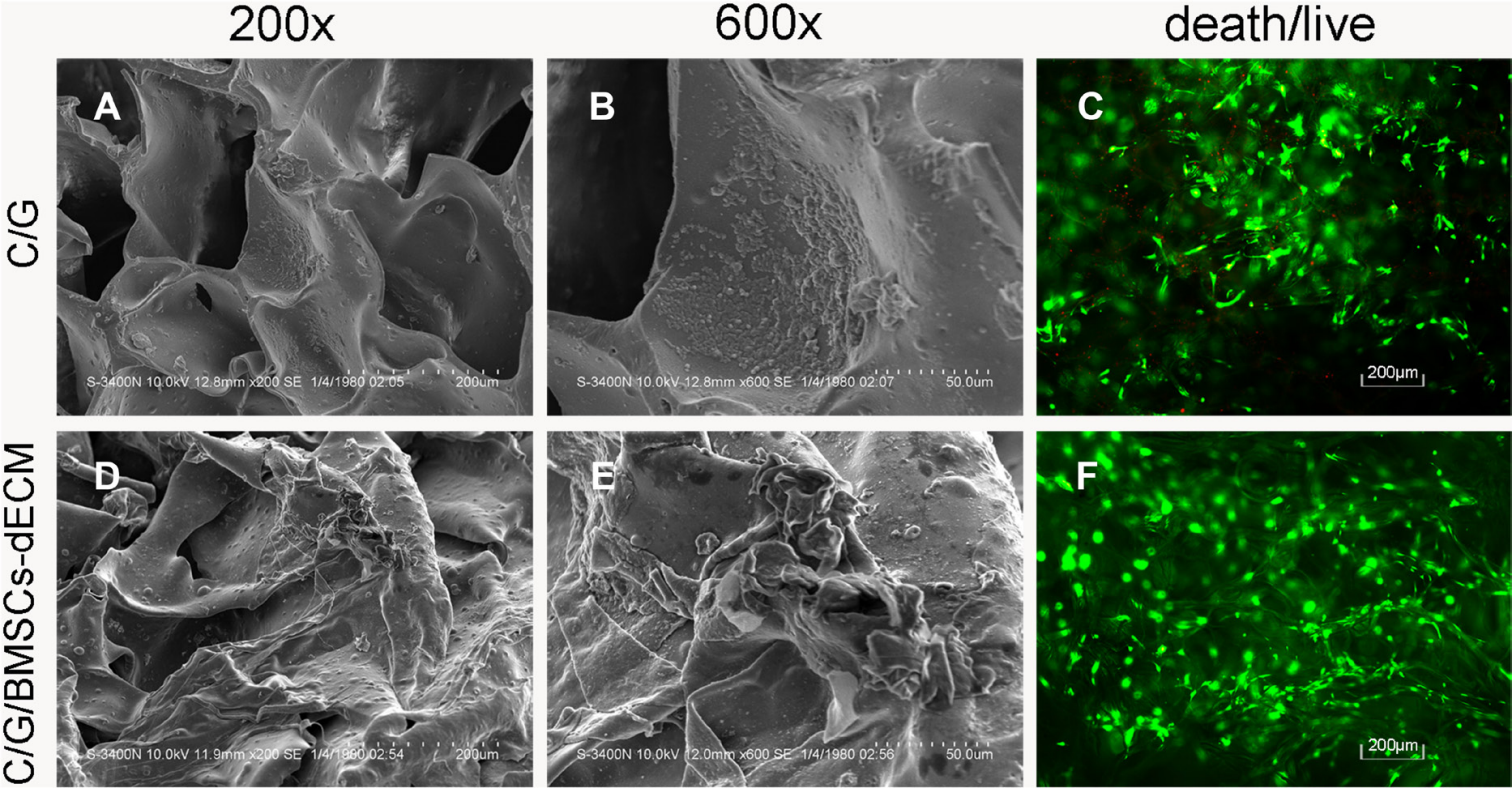
SEM and Death/live staining of cells/scaffolds SEM showed more ECM was secreted in C/G/BMSCs-dECM scaffolds (**D**, **E**) than C/G scaffolds (**A**, **B**). Death/live staining showed that almost no dead cells existed in C/G/BMSCs-dECM scaffolds (**F**), while some dead cells appeared in C/G scaffolds (**C**). A and D: ×200. B and E: ×600.

**Figure 4:**
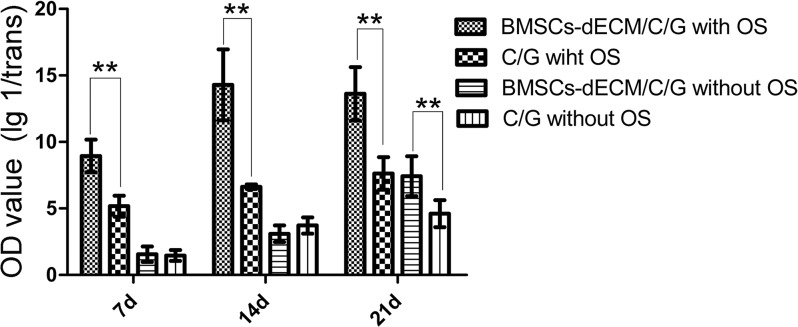
U values of ALP after 7 d, 14 d and 21 d Values are mean ± standard error of the mean (*n* = 6). ^**^P < 0.01, significant change with respect to control group.

The cell adhesion ratio of the C/G/BMSCs-dECM scaffolds was 54.17 ± 6.25%, while that of the C/G scaffolds was 40.00 ± 3.16%. After culturing for 7 days, live/dead staining showed that most cells in the C/G scaffolds and almost all of the cells in the C/G/BMSCs-dECM scaffolds were viable (Figure 3C and 3F); additionally, more live cells were observed in the C/G/BMSCs-dECM scaffolds (Figure 4F). Scanning electron microscopy(SEM) showed that cells in the C/G/BMSCs-dECM scaffolds (Figure 3D and 3E) were more active than cells in the C/G scaffolds (Figure 3A and 3B). Furthermore, these cells were well distributed, and the ECM secreted by them covered the walls of the C/G/BMSCs-dECM scaffolds (Figure 3D and 3E).

Figure 4 shows the alkaline phosphatase (ALP) results for cells in both scaffolds with and without osteogenic induction (OS). With OS, the U value of cells in the C/G/BMSCs-dECM scaffolds reached a high level after 7 days, peaked after 14 days and was maintained for 21 days. However, the U value of cells in the C/G scaffolds was much lower than that of the C/G/BMSCs-dECM scaffold cells and increased only slightly. Without OS, the U values of cells in the C/G/BMSCs-dECM and C/G scaffolds were similar after 7 and 14 days, but after 21 days, the U value of cells in the C/G/BMSCs-dECM scaffolds was higher than that of cells in the C/G scaffolds and was similar to that of cells in the C/G scaffolds with OS.

**Figure 5:**
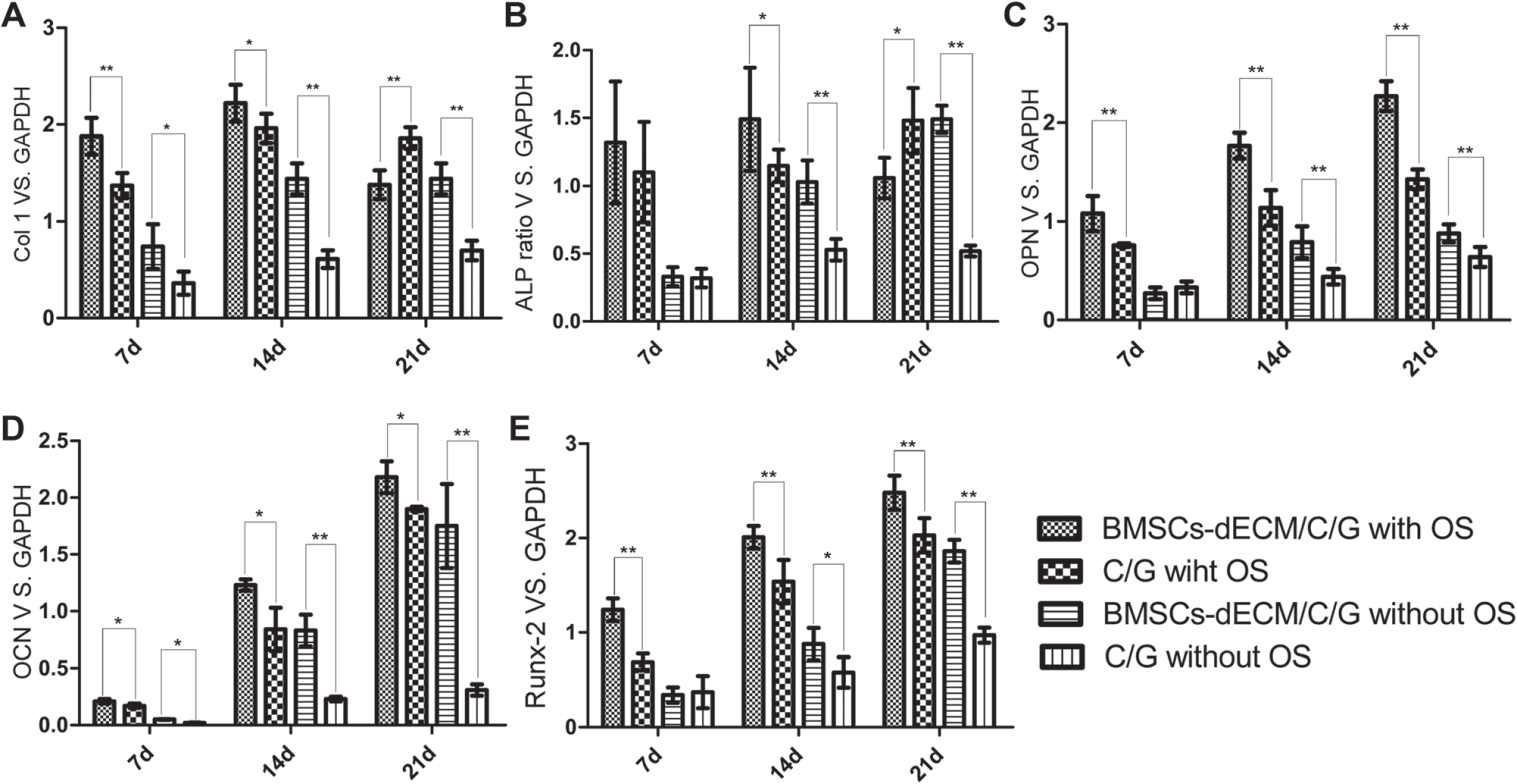
Osteogenic markers mRNA ratios vs. GAPDH after 7 d, 14 d and 21 d Values are mean ± standard error of the mean (*n* = 3). ^*^P < 0.05, ^**^P < 0.01, significant change with respect to control group.

Figure 5 shows the reverse transcription-polymerase chain reaction (RT-PCR) results for collagen 1 (Col 1), ALP, osteopontin (OPN), osteocalcin (OCN), and Runt-related transcription factor 2 (Runx-2) from cells in both scaffolds. The ALP messenger RNA(mRNA) trend was similar to that observed for the ALP protein levels. For Col 1 mRNA, with OS, the ratio reached a high level after 7 days, peaked after 14 days, then declined, and the Col 1 mRNA level of cells in C/G/BMSCs-dECM scaffolds was higher than that of cells in C/G scaffolds at day 7 and 14, while was lower than that at day 21; without OS, the Col 1 mRNA ratio of cells in C/G/BMSCs-dECM scaffolds was higher than that in C/G scaffolds except for day 7, where there was no statistic difference. With OS, the amount of OPN mRNA increased with time, and the OPN mRNA level of cells in the C/G/BMSCs-dECM scaffolds was higher than that of cells in the C/G scaffolds; without OS, the OPN mRNA ratios of cells in both scaffolds were much lower than those in cells with OS, and the level in C/G/BMSCs-dECM scaffolds was still higher than that of cells in the C/G scaffolds. For OCN mRNA, after 14 days, the ratio sharply increased and continued to increase until 21 days, and the ratio in the C/G/BMSCs-dECM scaffolds without OS was similar to the ratio in the C/G scaffolds with OS. For Runx-2 mRNA with or without OS, the ratio increased with time and peaked at day 21. Besides, The Runx-2 mRNA level of cells in C/G/BMSCs-dECM scaffolds was higher than that of cells in C/G scaffolds except for day 7 without OS.

**Figure 6:**
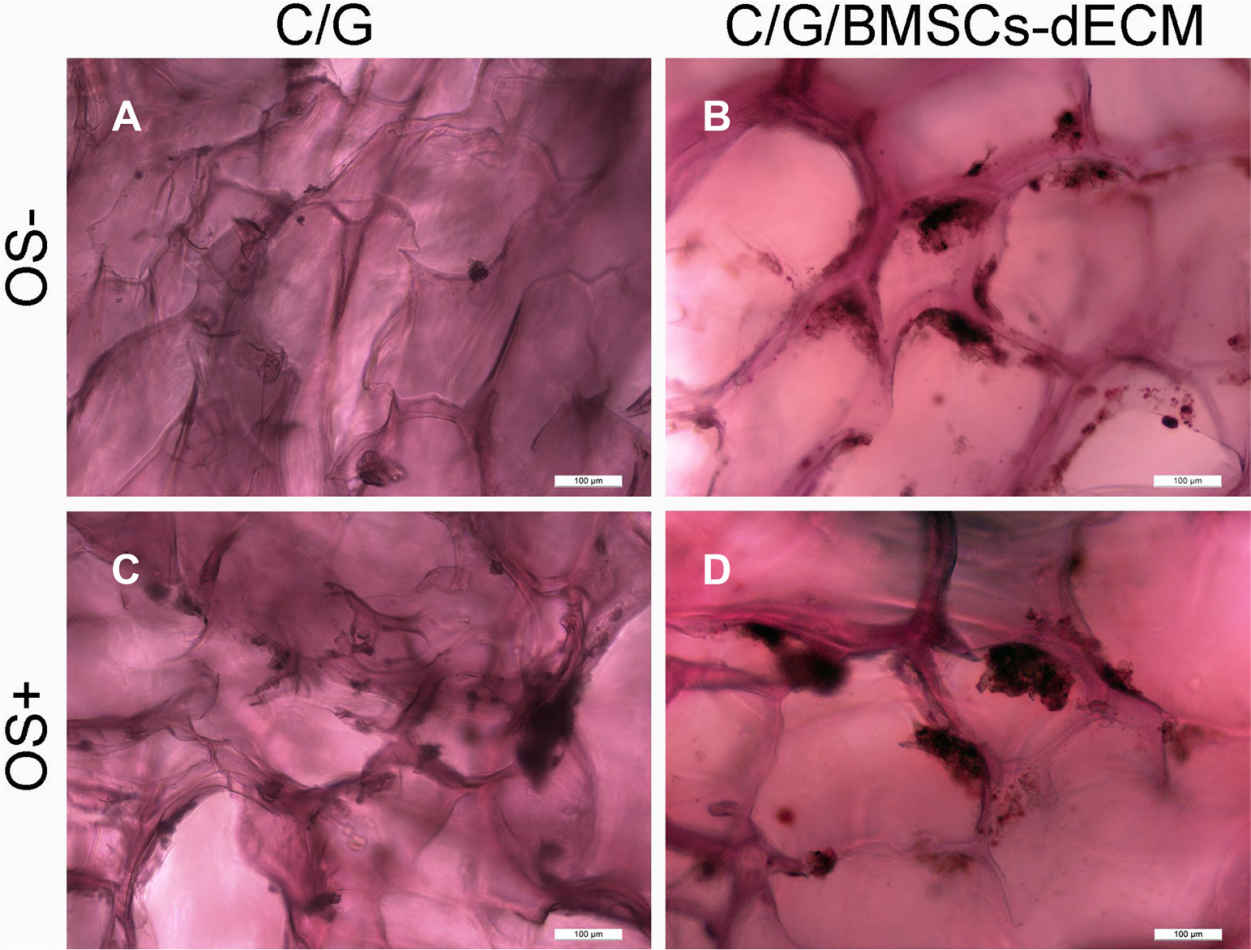
Alizarin red staining for calcium nodules After 21 days, C/G/BMSCs-dECM scaffolds (**B**, **D**) and C/G scaffolds (**A**, **C**) were stained with Alizarin red. (A, B) showd the results of both scaffolds without OS, while (C, D) showed the results of both scaffolds with OS.

**Figure 7:**
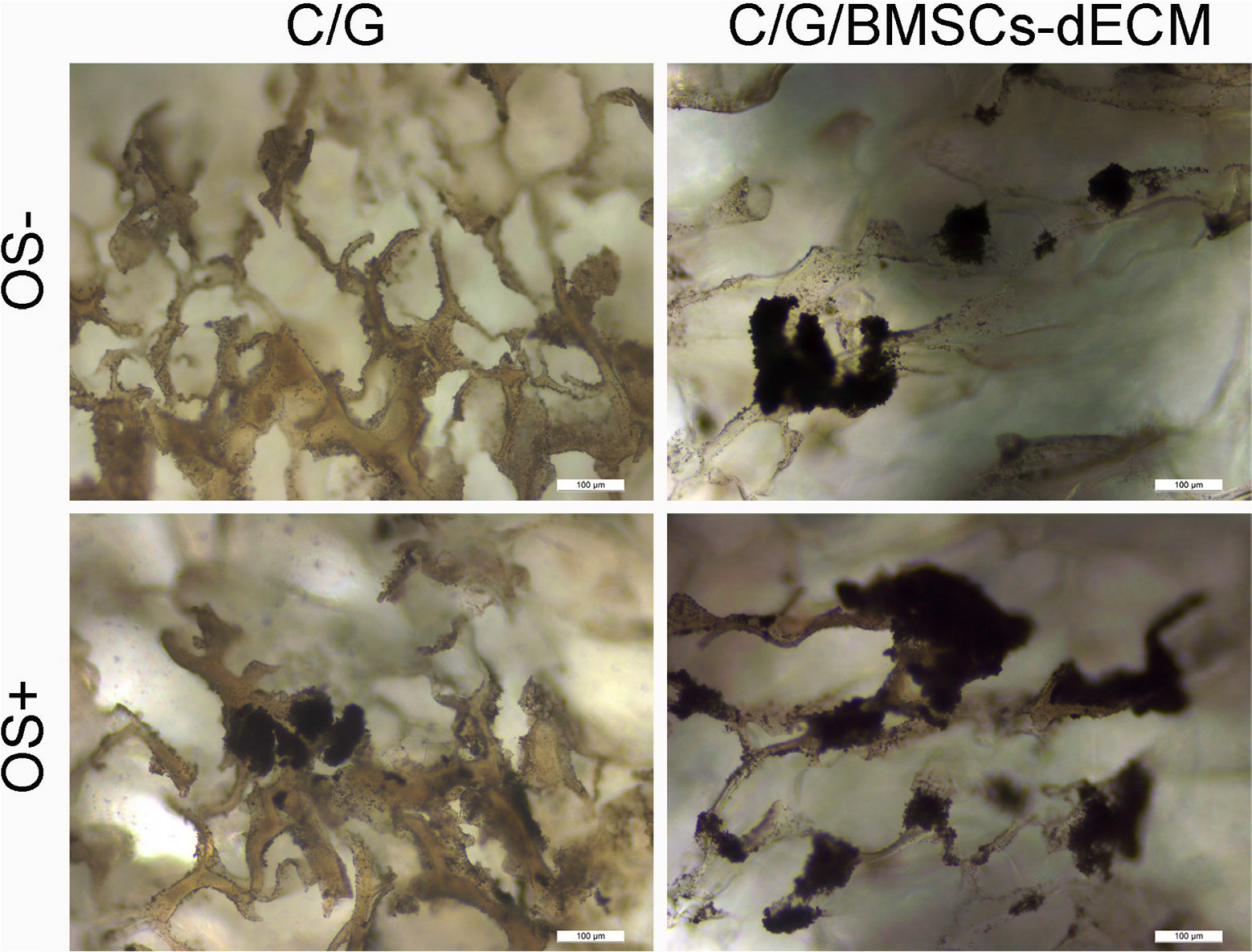
Von Kossa for calcium nodules After 21 days, C/G/BMSCs-dECM scaffolds (**B**, **D**) and C/G scaffolds (**A**, **C**) were stained with Von Kossa. (A, B) showd the results of both scaffolds without OS, while (C, D) showed the results of both scaffolds with OS.

Mineralization deposition was observed using alizarin red and von Kossa staining, and Figures 6 and 7 show that with OS, calcium nodules in the C/G/BMSCs-dECM scaffolds were thicker than those in the C/G scaffolds, especially with von Kossa staining; without OS, the nodules were smaller and more dispersed.

## DISCUSSION

A bioactive material that contains various growth factors and proteins, ECM has been used to fabricate tissue engineering scaffolds [13, 21–25]. With the method introduced by Liming Wang, we can obtain intact BMSCs-dECM; however, according to our previous experience, ECM suspensions exhibit problematic stickiness, and the powders obtained after lyophilization often do not form scaffolds. Moreover, even though scaffolds can be made by shortening the lyophilization time, their strength is poor. To the best of our knowledge, intact BMSCs-dECM scaffolds have never been applied to bone tissue engineering.

Chitosan is often used with gelatin [14, 16, 26]. Chitosan and gelatin solutions are very thick, and scaffolds other than powders can be obtained after lyophilization. Thus, in this study, we constructed C/G/BMSCs-dECM scaffolds and examined the feasibility of applying them to bone tissue engineering.

Both scaffold types were white and porous. A porous structure is beneficial for providing nutrition and allowing metabolic discharge from cells [27]. The pore size of the C/G/BMSCs-dECM scaffolds was larger than that of the C/G scaffolds (*p* < 0.05), but there was no significant difference in terms of their porosity (*p* > 0.05). As the ideal pore size is 100–350 μm [28], both scaffolds had a suitable pore size, which is necessary for cell adhesion and growth.

The water uptake ratio of the C/G/BMSCs-dECM scaffolds was higher than that of the C/G scaffolds, likely due to two factors. First, the larger pore size in the C/G/BMSCs-dECM scaffolds can lead to more widespread contact between the medium and the pore wall; second, hydrophilic groups in the ECM may promote water absorption [24], which provides two advantages. First, water absorption is beneficial for cell metabolism and providing nutrition, and second, after absorbing water, scaffolds swell, leading to larger scaffolds that come into closer contact with surrounding tissues, which avoids displacement of the implant [29].

For the C/G/BMSCs-dECM scaffolds, replacing 1% chitosan and gelatin with BMSCs-dECM led to decreased mechanical strength compared with that of the C/G scaffolds (*p* < 0.05). However, the compressive modulus of the C/G/BMSCs-dECM scaffolds was still much larger than that reported previously for ECM scaffolds [13].

Acetic acid and glutaraldehyde were used during the scaffold preparation process, which may affect cell growth (especially glutaraldehyde). Although chemical neutralization and flushing with double distilled water (ddw) were performed before the second lyophilization, we didnot know whether the reagents remained in the scaffolds. Therefore, we examined the toxicity of elutions from both scaffolds using CCK-8 assays and cell counts. When cells were cultured with the scaffold elutions, the cell proliferation ratios did not decrease. Moreover, the proliferation ratio in the presence of the 7-day elution from the C/G/BMSCs-dECM scaffolds was higher than that from the other scaffold elutions. These results indicated that there were no residual chemical reagents in either type of scaffold. Moreover, the C/G/BMSCs-dECM scaffolds likely slowly released bioactive factors into the medium that can accelerate cell proliferation, which requires further research.

The cell adhesion ratio of the C/G/BMSCs-dECM scaffolds was higher than that of the C/G scaffolds (*p* < 0.05), which is likely the result of two factors. First, more cells can interact with pore walls in the C/G/BMSCs-dECM scaffolds because of their larger pore size. Second, gelatins and proteins in the ECM, such as TGF-β_1_ which can recruit cells *in situ* [30], can provide a microenvironment for cell adhesion, proliferation and differentiation. When cultured for 7 days, live/dead cell staining and SEM were used to examine the viability and activity of the cells in both scaffolds. Cells in the C/G/BMSCs-dECM scaffolds were more active than those in the C/G scaffolds.

Osteogenic differentiation of BMSCs is a key process for bone repair and regeneration. Three different parameters, ALP levels, osteogenic markers and mineralization deposition, were assessed. With OS, ALP levels in the C/G/BMSCs-dECM scaffolds reached a high level after 7 days, peaked after 14 days, and remained stable for 21 days, while ALP levels in the C/G scaffolds exhibited a similar trend with a lower *U* value (*p* < 0.01). Without OS, ALP levels in the C/G/BMSCs-dECM scaffolds also reached a high level after 21 days that was similar to the level observed with OS in the C/G scaffolds (*p* > 0.05) and higher than that in the C/G scaffolds without OS (*p* < 0.05). These results indicated that C/G/BMSCs-dECM scaffolds displayed excellent osteogenic capabilities even without OS.

The osteogenic markers Col 1, ALP, OPN, OCN and Runx-2 were chosen to evaluate osteogenesis, because Col 1 is a early-term marker that means extracellular matrix appearance, ALP and OPN are early and medium-term osteogenic markers that are associated with extracellular matrix accumulation, and OCN is a late-term marker that indicates extracellular matrix maturity [18, 31, 32], which can cover the whole term of osteogenesis. Besides, Runx-2 is another important marker of osteogenic induction. Previous studies also selected several or all of these markers to assess the osteogenesis ability of stem cells [3, 23, 33, 34]. The results conformed to this temporal expression; Col1, ALP and OPN were high at 7 days and 14 days, while OCN levels were high at 14 days and 21 days.

Alizarin red and von Kossa staining were used to observe mineralization deposition. With OS, calcium nodules in the C/G/BMSCs-dECM scaffolds were larger and thicker than those in the C/G scaffolds. In addition, the von Kossa staining intensity appeared greater than that of alizarin red, likely for two reasons. First, alizarin red stained the scaffolds in addition to the calcium nodules; thus, we needed to flush the scaffolds several times to remove the residual dye, which led to the loss of calcium nodules. Second, different methods and durations of staining can produce disparate results and may have also led to the differences we perceived. Without OS, we also observed calcium nodules in both of the scaffolds, and the calcium nodules in the C/G/BMSCs-dECM scaffolds were likewise larger than those in the C/G scaffolds. Given the ALP, osteogenic markers and mineralization deposition results, we conclude that BMSCs-dECM and C/G/BMSCs-dECM scaffolds are promising materials for bone tissue engineering.

There are some limitations to our study. First, we could not quantify the fluorescence intensities nor the calcium nodules due to the multilayered structure of the scaffolds, which may have led to bias in the results. Second, there were differences between the *in vivo* and *in vitro* studies; thus, further *in vivo* research is needed to verify the *in vitro* results.

In conclusion, BMSCs-dECM is a promising material for use in bone tissue engineering, as is the C/G/BMSCs-dECM scaffold that was fabricated from it.

## MATERIALS AND METHODS

### Rats

Male Sprague-Dawley (S-D) rats (weighing approximately 50 g) were purchased from the animal center at the 2nd Affiliated Hospital of Harbin Medical University. All animal experiments were performed in compliance with the Institutional Animal Care and Use Committee of Harbin Medical University.

### Isolation and identification of BMSCs

BMSCs were isolated according to a previously described method [13]. Briefly, an approximately 50g S-D rat was euthanized by pentobarbital overdose. Both femurs were removed, and the muscles were cleaned under sterile conditions. The bone marrow was flushed out with Dulbecco's modified Eagle's medium (DMEM; Beyotime, Shanghai, China), and the resulting DMEM/bone marrow mixture was centrifuged. After discarding the supernatant, the sediment was resuspended in complete medium, comprising DMEM, 10% fetal bovine serum (FBS) and 1% penicillin/streptomycin (Beyotime, Shanghai, China). The cells were then cultured in an incubator(5%CO_2_, 37°C, 95% humidity). After 3 days, the non-adherent cells were removed, and the medium was refreshed every 2 days until the cells reached 90–100% confluence. The cells were then passaged, and after the 3rd passage, the cell phenotype was verified as described in our previous study [3]. These cells were found to meet the criteria for mesenchymal stem/stromal cells [35].

### Collection of BMSCs-dECM

**Figure 8:**
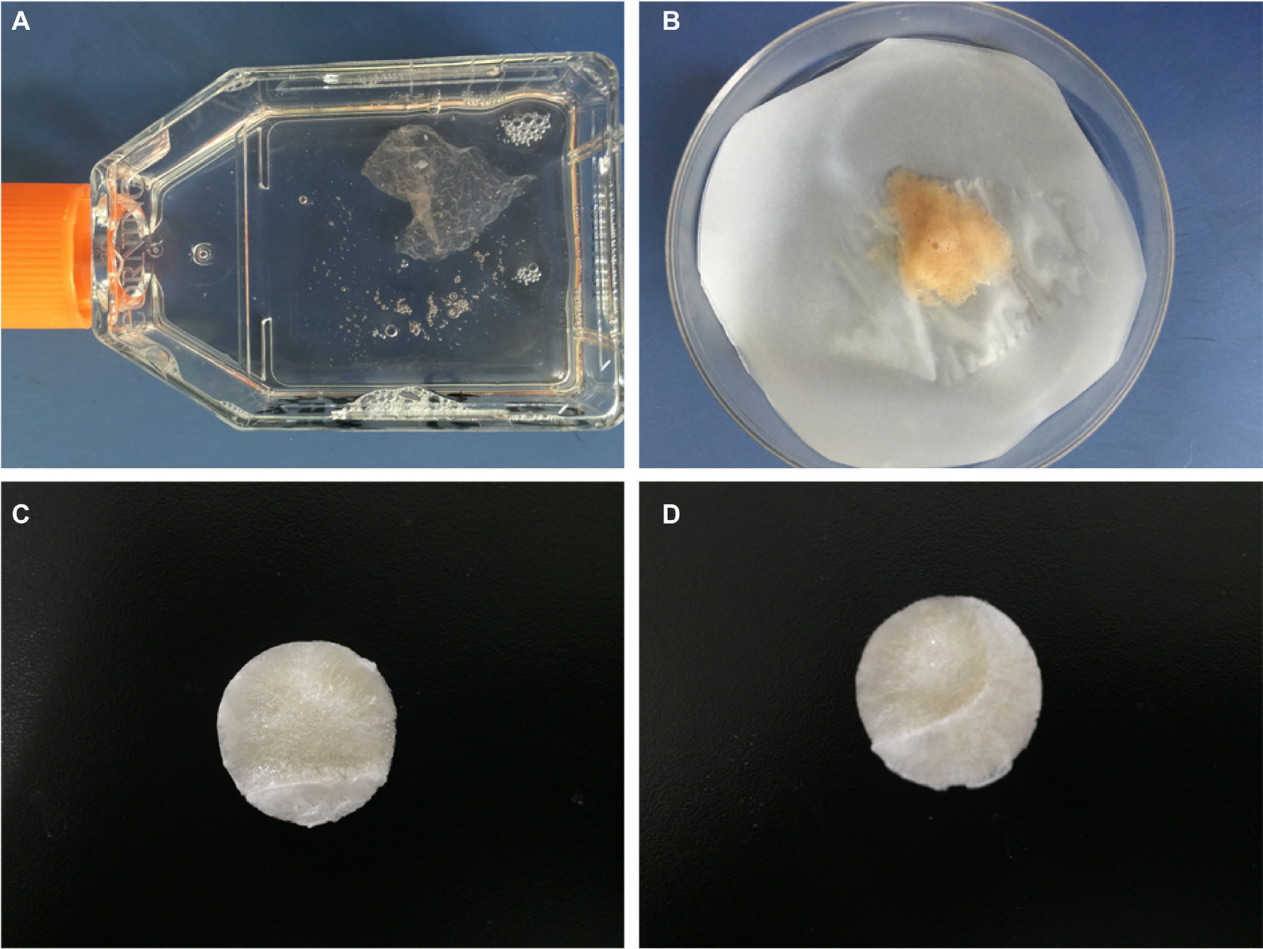
Preparation of scaffolds After being cultured 3 weeks, gel-like ECM appeared (**A**), which showed faint yellow when collected (**B**). After suspended, lyophilized and crosslinked, C/G/BMSCs-dECM scaffolds (**C**) and C/G scaffolds (**D**) were obtained, which both were white and porous.

BMSCs-dECM was collected as described previously [13]. Briefly, cells from the 3rd passage were cultured in a bottle containing DMEM supplemented with 50 μg/mL L-ascorbic acid and 150 μg/mL ascorbate-2-phosphate. The medium was refreshed every 3 days for 4 weeks. Gel-like ECM appeared on the bottom surface of the bottle. We then discarded the medium, patted the bottom of bottle and added 2 mL of trypsin for 2 min (37°C, 5% CO_2_, 95% humidity) to remove any cells on the ECM membrane (Figure 8A), and the ECM was collected (Figure 8B). The BMSCs-dECM was subsequently lyophilized and stored at −80°C for further use.

### Preparation of C/G/BMSCs-dECM and C/G scaffolds

Chitosan(Biosharp,Anhui,China) was dissolved in acetic acid (1%v/v), and gelatin (Biosharp, Anhui, China) was dissolved in ddw in a 55°C water bath; A BMSCs-dECM suspension was prepared with a tissue homogenizer. These three materials were mixed in proper proportions to yield a mixture containing 2% w/v chitosan, 2% w/v gelatin and 1% w/v BMSCs-dECM. The mixture components were then cross-linked using 0.25% glutaraldehyde (Tian li, Tianjin, China) overnight. A 12-well plate was used as a lyophilization mold for the scaffolds. The mixed solution was added to the wells, incubated at −80°C for 8 h and then lyophilized for 48 h. White and porous scaffolds were obtained, which were then soaked in 1% w/v NaOH (Tian li, Tianjin, China) for 30 min, flushed with ddw, soaked in 2% sodium borohydride (Tian li, Tianjin, China) for 20 min and flushed with ddw to remove the acetic acid and glutaraldehyde. Next, a second lyophilization was performed to produce the final scaffolds (Figure 8C).

C/G scaffolds (Figure 8D), which contained 2.5% chitosan and 2.5% gelatin, were also constructed according to the above method. Both scaffolds were sterilized with ethylene oxide before further use.

### Basic characteristics

The color, shape, and basic structure of the scaffolds were examined by visual inspection. Hematoxylin andeosin (H&E) staining and SEM were used to observe their internal structures. Briefly, 5-μm slices were prepared as described in our previous study and subjected to H&E staining [3]. The scaffolds were coated with gold/palladium, and their morphology was visualized via SEM. Under appropriate magnification, 10 pores from 3 random views were randomly selected and measured, and their average value was determined to be the scaffold pore size [36].

### Porosity

Porosity was measured according to a previous report [37]. Briefly, scaffolds were cut into approximately 1 cm^3^ cubes. For each cube, the length, width and height were accurately measured using a digital caliper; an accurate volume (Vw) was calculated; and their weight (Ws) was measured. Each scaffold cube was then immersed in a container full of ethanol (ρ), and the displaced ethanol was collected in another container. Next, we measured the weight (W1) of the mixture containing the scaffold, ethanol and both containers. After removing the displaced ethanol, we measured the weight (W2) of the mixture containing the scaffold, the remaining ethanol and both containers. The porosity was calculated as 1-(W1−W2 + Ws)/(ρ × Vw) × 100%.

### Water uptake ratio

Each scaffold (W1) was immersed in ddw for 2 h and then weighed (W2) again. The water uptake ratio was calculated as (W2−W1)/W1.

### Mechanical properties

According to a previously reported method, scaffold samples that were 6 mm in diameter and 2 mm in thickness were loaded onto a universal mechanical tester. A 10N load cell was used, and the scaffolds were compressed at 50N/min until they could no longer withstand the pressure. The compressive press-strain curve was linear up to a strain rate of 50%, and the compressive modulus was at 50%.

### Cytotoxicity

To test their *in vitro* cytotoxicity, we immersed scaffolds in 5 mL of complete medium and cultured them at 4°C for 1, 3 and 7 days. We then removed the scaffolds and collected the culture mixtures containing complete medium and the scaffold elutions. BMSCs from the 3rd passage were cultured in a 96-well plate at a density of 1 × 10^4^ cells/well with complete medium and six different mixtures of the Day 1 elutions, Day 3 elutions and Day 7 elutions of the C/G/BMSCs-dECM and C/G scaffolds. After 3 days of incubation, CCK-8 (Jian Cheng, Nanjing, China) was used to measure the absorbance (OD value) of the cultures according to the manufacturer's instructions. With this method, higher absorbance values indicated better activity. In addition, the final number of cells (n_1_) per well was determined by plate counts, and the proliferation ratio was calculated as n_1_/1 × 10^4^. The cell viability and proliferation ratios were used to assess the cytotoxicity of both types of scaffolds.

### Adhesion ratio

A dynamic method was employed to test the cell adhesion ratio. Briefly, scaffolds were immersed in 5 mL of complete medium containing 1 × 10^5^ 3rd passage cells. The mixture was subjected to vibration overnight on a shock table, during which a portion of the cells adhered to the scaffolds. The scaffolds with adhered cells were removed and further cultured, and the non-adherent cells and cells that adhered to the well walls (n_1_) were collected and counted. The adhesion ratio was calculated as (1 × 10^5^-n_1_)/1 × 10^5^ × 100%.

### Cytoactivity

Scaffolds with adhered cells were further cultured in complete medium for 7 days, and live/dead cell staining was employed to assess cell activity in the scaffolds. Briefly, a live/dead cell staining kit (Tian Kai,Tianjin,China) composed of two reagents, Calcein AM and EthD-1, was used. Calcein AM enters live cells and is then hydrolyzed by a special enzyme to calcein, which is a green fluorescent molecule. EthD-1 cannot enter living cells, but it can enter dead cells, where it combines with gene segments to form a fluorescent signal, causing dead cells to appear red. SEM was also performed to analyze the morphology and activity of cells in the scaffolds.

### Osteogenic induction

After being removed, scaffolds with adhered cells were cultured with and without osteogenesis induction medium (Cyagen,Guangdong,China), and these medium were refreshed every 3 days. After 7, 14 and 21 days, ALP levels and mineralization deposition were measured and the mRNA levels of Col 1, ALP, OPN, OCN and Runx-2 were determined to evaluate cell osteogenesis in the different scaffolds under different situations.

For ALP (Jian Cheng, Nanjing, China), the scaffolds with adhered cells were immersed in 3 mL of trypsin for 2 min with light shaking, and then the trypsin was neutralized with complete medium. The scaffolds were removed, and the remaining mixture was collected. After centrifugation and resuspension, the cells were obtained. Ultrasound was employed to disrupt the cell membranes, and ALP assays were performed according to the manufacturer's instructions. OD and U values were measured and calculated. This procedure was repeated 3 times.

For RT-PCR, the cells were extracted from the scaffolds as outlined above. TRIzol reagent was used for total RNA isolation, and cDNA was synthesized using a Golden 1st cDNA Synthesis Kit. Real-time PCR assays for mRNA were performed in a Mini-Opticon2 system (MJ) using the Golden HS SYBR Green qPCR Mix with the following reaction conditions: 95°C for 15 min, followed by 45 cycles of 95°C for 10 s and 60°C for 30 s. Primers for Col 1, ALP, OPN, OCN and Runx-2 were purchased from Genscript, and the GAPDH qPCR primers were obtained from HaiGene (Table 2). Quantitative normalization of the cDNA in each sample was performed using the GAPDH gene as an internal control. RT-PCR assays were performed in triplicate for each sample, and the mean values were used to calculate the mRNA expression levels.

**Table 2:** Primers for RT-PCR

GENE	Primer sequences
Runx2 -F	5′-CTTTCTTCGTTGTCTGTTTACTTC-3′
Runx2-R	5′-GTTGTTGTTGTTGTTGTTGTTCC-3′
ALP-F	5′-GTGTGTGCTGACTGTAACCTC-3′
ALP -R	5′-CGTGAATGAGTGTTCTCGTCTG-3′
OPN -F	5′-ACGATGATGACGACGACGATG -3′
OPN –R	5′-TTGTGTGCTGGCAGTGAAGG-3′
OCN -F	5′-CAGTAAGGTGGTGAATAGACTCCG-3′
OCN –R	5′-GGTGCCATAGATGCGCTTG-3′
Col 1-F	5′-CTGAGATGCTCCCTAGACC-3′
Col 1-R	5′-CCCTTGTTAAATAGCACCTTC-3′
Rat GAPDH-F	5′-AACTCCCATTCTTCCACCTTT-3′
Rat GAPDH-R	5′-CTCTTGCTCTCAGTATCCTTG-3′

Alizarin red and von Kossa staining were used to analyze mineralization deposition. Briefly, after being cultured for 21 days with and without OS, scaffolds with adhered cells were immersed in 4% paraformaldehyde overnight. For alizarin red staining, the scaffolds were immersed in alizarin red for 3 min and then flushed with ddw several times to remove residual dye. For von Kossa staining, the scaffolds were immersed in 5% von Kossa solution, exposed to ultraviolet light for 10 min, and flushed with ddw several times to remove residual dye. Finally, the scaffolds were observed under an inverted microscope.

### Statistical analysis

All examinations were performed on six replicate scaffolds unless otherwise indicated, and the data are presented as the mean ± SD. Statistical differences were analyzed using Mann-Whitney *U* test or Kruskal-Wallis ANOVA followed by a Nemenyi test, and *p* values of < 0.05 or < 0.01 were considered significant.

## Abbreviations

ECM: extracellular matrix
C/G: chitosan/gelatin
BMSCs: bone marrow stem cells
BMSCs-dECM: bone marrow stem cells-derived extracellular matrix
VEGF: vascular endothelial growth factor
TGF-β1: transforming growth factor beta-1
DMEM: Dulbecco's modified Eagle's medium
FBS: fetal bovine serum
ddw: double distilled water
H&E: Hematoxylin andeosin
SEM: scanning electron microscopy
OD: absorbance
ALP: alkaline phosphatase
RT-PCR: reverse transcription-polymerase chain reaction
OS: osteogenic induction
OPN: osteopontin
OCN: osteocalcin
Col 1: collagen 1
Runx-2: Runt-related transcription factor 2

## References

[1] Qi X, Pei P, Zhu M, Du X, Xin C, Zhao S, Li X, Zhu Y (2017). Three dimensional printing of calcium sulfate and mesoporous bioactive glass scaffolds for improving bone regeneration *in vitro* and *in vivo*. Sci Rep.

[2] Fu TS, Chang YH, Wong CB, Wang IC, Tsai TT, Lai PL, Chen LH, Chen WJ (2015). Mesenchymal stem cells expressing baculovirus-engineered BMP-2 and VEGF enhance posterolateral spine fusion in a rabbit model. Spine J.

[3] Wang X, Yu T, Chen G, Zou J, Li J, Yan J (2017). Preparation and Characterization of a Chitosan/Gelatin/Extracellular Matrix Scaffold and Its Application in Tissue Engineering. Tissue Eng Part C Methods.

[4] Midwood KS, Williams LV, Schwarzbauer JE (2004). Tissue repair and the dynamics of the extracellular matrix. Int J Biochem Cell Biol.

[5] Reilly GC, Engler AJ (2010). Intrinsic extracellular matrix properties regulate stem cell differentiation. J Biomech.

[6] Bhrany AD, Beckstead BL, Lang TC, Farwell DG, Giachelli CM, Ratner BD (2006). Development of an esophagus acellular matrix tissue scaffold. Tissue Eng.

[7] Crapo PM, Gilbert TW, Badylak SF (2011). An overview of tissue and whole organ decellularization processes. Biomaterials.

[8] Flynn LE, Prestwich GD, Semple JL, Woodhouse KA (2008). Proliferation and differentiation of adipose-derived stem cells on naturally derived scaffolds. Biomaterials.

[9] Cheng HW, Tsui YK, Cheung KM, Chan D, Chan BP (2009). Decellularization of chondrocyte-encapsulated collagen microspheres: a three-dimensional model to study the effects of acellular matrix on stem cell fate. Tissue Eng Part C Methods.

[10] Choi KH, Choi BH, Park SR, Kim BJ, Min BH (2010). The chondrogenic differentiation of mesenchymal stem cells on an extracellular matrix scaffold derived from porcine chondrocytes. Biomaterials.

[11] Liao J, Guo X, Grande-Allen KJ, Kasper FK, Mikos AG (2010). Bioactive polymer/extracellular matrix scaffolds fabricated with a flow perfusion bioreactor for cartilage tissue engineering. Biomaterials.

[12] Wolchok JC, Tresco PA (2010). The isolation of cell derived extracellular matrix constructs using sacrificial open-cell foams. Biomaterials.

[13] Xu Y, Xu GY, Tang C, Wei B, Pei X, Gui JC, Min BH, Jin CZ, Wang LM (2015). Preparation and characterization of bone marrow mesenchymal stem cell-derived extracellular matrix scaffolds. J Biomed Mater Res B Appl Biomater.

[14] Li J, Yang B, Qian Y, Wang Q, Han R, Hao T, Shu Y, Zhang Y, Yao F, Wang C (2015). Iota-carrageenan/chitosan/gelatin scaffold for the osteogenic differentiation of adipose-derived MSCs in vitro. J Biomed Mater Res B Appl Biomater.

[15] Miranda SC, Silva GA, Mendes RM, Abreu FA, Caliari MV, Alves JB, Goes AM (2012). Mesenchymal stem cells associated with porous chitosan-gelatin scaffold: a potential strategy for alveolar bone regeneration. J Biomed Mater Res A.

[16] Isikli C, Hasirci V, Hasirci N (2012). Development of porous chitosan-gelatin/hydroxyapatite composite scaffolds for hard tissue-engineering applications. J Tissue Eng Regen Med.

[17] Miranda SC, Silva GA, Hell RC, Martins MD, Alves JB, Goes AM (2011). Three-dimensional culture of rat BMMSCs in a porous chitosan-gelatin scaffold: A promising association for bone tissue engineering in oral reconstruction. Arch Oral Biol.

[18] Wang K, Bruce A, Mezan R, Kadiyala A, Wang L, Dawson J, Rojanasakul Y, Yang Y (2016). Nanotopographical Modulation of Cell Function through Nuclear Deformation. ACS Appl Mater Interfaces.

[19] Song L, Wang K, Li Y, Yang Y (2016). Nanotopography promoted neuronal differentiation of human induced pluripotent stem cells. Colloids Surf B Biointerfaces.

[20] Yang Y, Wang K, Gu X, Leong KW (2017). Biophysical Regulation of Cell Behavior—Cross Talk between Substrate Stiffness and Nanotopography. Engineering.

[21] Li J, Sun H, Zhang R, Li R, Yin Y, Wang H, Liu Y, Yao F, Yao K (2010). Modulation of mesenchymal stem cells behaviors by chitosan/gelatin/pectin network films. J Biomed Mater Res B Appl Biomater.

[22] Tang C, Jin C, Xu Y, Wei B, Wang L (2016). Chondrogenic Differentiation Could Be Induced by Autologous Bone Marrow Mesenchymal Stem Cell-Derived Extracellular Matrix Scaffolds Without Exogenous Growth Factor. Tissue Eng Part A.

[23] Kang Y, Kim S, Bishop J, Khademhosseini A, Yang Y (2012). The osteogenic differentiation of human bone marrow MSCs on HUVEC-derived ECM and beta-TCP scaffold. Biomaterials.

[24] Gilbert TW, Sellaro TL, Badylak SF (2006). Decellularization of tissues and organs. Biomaterials.

[25] Tang C, Xu Y, Jin C, Min BH, Li Z, Pei X, Wang L (2013). Feasibility of autologous bone marrow mesenchymal stem cell-derived extracellular matrix scaffold for cartilage tissue engineering. Artif Organs.

[26] Mao J, Zhao L, De Yao K, Shang Q, Yang G, Cao Y (2003). Study of novel chitosan-gelatin artificial skin *in vitro*. J Biomed Mater Res A.

[27] Ji C, Annabi N, Khademhosseini A, Dehghani F (2011). Fabrication of porous chitosan scaffolds for soft tissue engineering using dense gas CO2. Acta Biomater.

[28] Whang K, Healy KE, Elenz DR, Nam EK, Tsai DC, Thomas CH, Nuber GW, Glorieux FH, Travers R, Sprague SM (1999). Engineering bone regeneration with bioabsorbable scaffolds with novel microarchitecture. Tissue Eng.

[29] Zhang K, Zhang Y, Yan S, Gong L, Wang J, Chen X, Cui L, Yin J (2013). Repair of an articular cartilage defect using adipose-derived stem cells loaded on a polyelectrolyte complex scaffold based on poly(l-glutamic acid) and chitosan. Acta Biomater.

[30] Mcdevitt CA, Wildey GM, Cutrone RM (2003). Transforming growth factor-beta1 in a sterilized tissue derived from the pig small intestine submucosa. J Biomed Mater Res A.

[31] Osyczka AM, Leboy PS (2005). Bone morphogenetic protein regulation of early osteoblast genes in human marrow stromal cells is mediated by extracellular signal-regulated kinase and phosphatidylinositol 3-kinase signaling. Endocrinology.

[32] Zou J, Yuan C, Wu C, Cao C, Yang H (2014). The effects of platelet-rich plasma on the OS of bone marrow mesenchymal stem cells. Connect Tissue Res.

[33] Lin H, Yang G, Tan J, Tuan RS (2012). Influence of decellularized matrix derived from human mesenchymal stem cells on their proliferation, migration and multi-lineage differentiation potential. Biomaterials.

[34] Bae SE, Bhang SH, Kim BS, Park K (2012). Self-assembled extracellular macromolecular matrices and their different osteogenic potential with preosteoblasts and rat bone marrow mesenchymal stromal cells. Biomacromolecules.

[35] Reyes M, Lund T, Lenvik T, Aguiar D, Koodie L, Verfaillie CM (2001). Purification and ex vivo expansion of postnatal human marrow mesodermal progenitor cells. Blood.

[36] Yang F, Qu X, Cui W, Bei J, Yu F, Lu S, Wang S (2006). Manufacturing and morphology structure of polylactide-type microtubules orientation-structured scaffolds. Biomaterials.

[37] Spiller KL, Liu Y, Holloway JL, Maher SA, Cao Y, Liu W, Zhou G, Lowman AM (2012). A novel method for the direct fabrication of growth factor-loaded microspheres within porous nondegradable hydrogels: controlled release for cartilage tissue engineering. J Control Release.

